# Surveillance of Human Calicivirus in Spain

**DOI:** 10.3201/eid1108.041166

**Published:** 2005-08

**Authors:** Alicia Sánchez-Fauquier, Isabel Wilhelmi, Enriqueta Roman, Javier Colomina, Vanessa Montero, Ana Negredo

**Affiliations:** * Instituto De Salud Carlos III, Madrid, Spain;; † Hospital Severo Ochoa, Madrid, Spain;; ‡ Hospital De Fuenlabrada, Madrid, Spain;; § Hospital De La Ribera, Valencia, Spain

**Keywords:** Gastroenteritis, Noroviruses, Outbreak, Genotype diversity, Sporadic cases

**To the Editor:** Human caliciviruses (HuCVs) are an important cause of acute viral gastroenteritis in young children worldwide (1,2). In Spain, norovirus infections are not subject to specific surveillance; few data exist about sporadic cases (3,4) and none about outbreaks across the country. We have conducted a surveillance study of acute gastroenteritis epidemics to determine the prevalence of HuCV infections. Our goal was to gain insight into the epidemiology of these infections in Spain and consider new directions to prevent them and control improvements in food and water quality and sanitary practices.

From October 1998 to October 1999, a pilot prospective program was designed to study viral strains causing annual epidemics of severe diarrhea in children. A total of 822 stool specimens were obtained from children <4 years of age with sporadic gastroenteritis, who visited the emergency room of a hospital in Madrid. A gastroenteritis episode was defined as ≤3 looser-than-normal stools within a 24-h period. Clinical and epidemiologic information was collected. No pathogens were detected in fecal specimens from 292 children. A subset of 201 of these samples was tested for HuCVs.

Additionally, 741 fecal samples were collected from 135 outbreaks that occurred throughout Spain (13 of 17 geographic areas) from 2000 to 2002. An outbreak was defined with Kaplan criteria (5). Epidemiologic data (mode of transmission, setting and size of the outbreak, persons affected, persons at risk, attack rate, and age of affected persons) were recorded on a standardized form submitted to the National Microbiology Center.

Enteropathogenic bacteria in fecal specimens were examined by conventional culture procedures. Viruses (group A rotavirus, adenovirus, and astrovirus) were also examined by commercial enzyme immunoassay (Dako Diagnostics, Cambridgeshire, UK). In negative samples (1,033 of 1,563), viral RNA was extracted as described (3) and analyzed by reverse transcription–polymerase chain reaction (RT-PCR) for HuCVs by using JV12/JV13 primers (6). RT-PCR–negative samples were also reanalyzed with p289/290 (7) and NVp110-Nvp69 (8) primers pair, which detect sapovirus. HuCV-positive specimens were confirmed and genetically characterized by the reverse line blot hybridization method (9) and sequencing assays by using High Pure PCR Product Purification kit (Boehringer, Mannheim, Germany) and ABI PRISM BigDye Terminator Cycle Sequencing Reaction Kit (Perkin Elmer Biosystems, Foster City, CA, USA) on an automated sequencer (Applied Biosystems model 3700). For HuCV phylogenetic analyses, a multiple-sequence alignment was generated from the consensus sequence of each of the isolates and the reference strains by using ClustalX 1.8 methods. The nucleotide sequences were analyzed with the MEGA 2.1 analytical package by using neighbor-joining methods and Kimura 2-parameters algorithm. GenBank accession numbers for sequences described in this study are AY207341–AY207365.

In pediatric gastroenteritis cases, HuCVs were detected by RT-PCR in 63 specimens (31%). Twenty-nine of these were genotyped, and results of phylogenetic analyses are shown in the Table. The median age of children with HuCV infection was 15 months (range 1–47). In HuCV-positive cases, 83% were associated with vomiting, 32% with fever, 24% with mild dehydration, and 2% with severe dehydration. Hospitalization was required in 13% of the cases.

**Table:** Human caliciviruses (HuCVs) and phylogenetic clusters found in sporadic pediatric cases and gastroenteritis outbreak

	Pediatric cases (N = 201) (%)	Outbreak incident (N = 135) (%)
HuCVs found by reverse transcription–polymerase chain reaction	63 (31)	85 (63)
HuCVs analyzed phylogenetically	29	83
Sapovirus	3 (10)	0
Norovirus*	26 (90)	85 (100)
GI-Desert Shield cluster	0	5
GI-Queens Arms cluster	1	1
GII-Hawaii cluster	0	8
GII-Leeds cluster	0	1
GII-Lordsdale cluster	23	63
GII-Meklsham cluster	0	5
GII-Mexico cluster	2	0

*GI, genogroup I; GII, genogroup II.

Additionally, noroviruses were detected in 85 (63%) of 135 outbreaks. Seventy- seven (91%) of them were segregated into genogroup II (Table). Detection rates within outbreaks ranged from 13% to 100%. The setting was provided for 82 (97%) of 85 norovirus outbreaks. Nursing homes (57%) were the most common setting, followed by schools (10%), camping and vacation destinations (7%), hospitals (6%), and restaurants and hotels (4% each). The attack rate was provided for 33 (39%) norovirus outbreaks, and the median number of persons affected was 35. The mode of transmission was provided for 30 (35%) of the norovirus-positive outbreaks; the most common mode of transmission was person-to-person contact (n = 15), followed by contaminated food (n = 10) and contaminated water (n = 5).

This report shows the importance and diversity of HuCVs circulating throughout Spain. Noroviruses particularly have been found as a main causative agent of sporadic pediatric cases and outbreaks. The lower prevalence of sapovirus is similar to that shown by other authors (2), perhaps because sapovirus causes milder symptoms than norovirus. Analysis showed the predominance of norovirus genogroup II and Lordsdale cluster as the main genotypes both in sporadic cases and outbreaks, also shown in other reports (1,2,6).

Our study confirms that noroviruses are the main cause of nonbacterial gastroenteritis outbreaks throughout Spain, as in other European countries (1,10). However, we consider that HuCV infections could be underdiagnosed because a substantial number of nonbacterial outbreaks are labeled of unknown etiology. The systematic application of sensitive techniques to detect these viruses, as well as a more systematic surveillance system for viral diarrhea, would provide broader knowledge of norovirus infection in Spain.
